# Cheilitis Granulomatosa: A Case Report of a Sarcoid Mimic

**DOI:** 10.7759/cureus.80879

**Published:** 2025-03-20

**Authors:** Stephanie Nagy, Marika Fraser, Marc M Kesselman

**Affiliations:** 1 Department of Rheumatology, Kiran C. Patel College of Osteopathic Medicine, Nova Southeastern University, Davie, USA; 2 Division of Otolaryngology, Memorial Healthcare System, Hollywood, USA

**Keywords:** cheilitis granulomatosa, granulomatous cheilitis, lip swelling, melkersson-rosenthal syndrome, miescher cheilitis, orofacial granulomatosis, sarcoid mimicker

## Abstract

Cheilitis granulomatosa (CG) is a persistent and progressive swelling of the lips that can be non-tender and soft or firm to touch, with noncaseating granulomas that are perilymphatic and may show intralymphatic histiocytosis and lymphatic dilatation. CG can occur as an isolated condition or as part of Melkersson-Rosenthal syndrome, which also includes facial paralysis and a fissured tongue. The etiology of CG is currently unknown but has been hypothesized to be connected to genetics, allergies, immunological processes, and infectious causes. This case provides further evidence for the pathogenic causes of CG. We present a 46-year-old male patient with four years of progressive lip swelling to an outpatient rheumatological clinic, the cause of which has yet to be determined following numerous visits to healthcare providers. Corticosteroids were previously attempted, resulting in minor improvements in swelling; however, following discontinuation, the lip swelling returned. Laboratory findings were significant for Saccharomyces and Lyme disease, while other autoimmune biomarkers remained negative, and a biopsy indicated noncaseating granulomas, leading to the diagnosis of CG. The patient was started on mycophenolate, and following treatment, there was a significant reduction in the swelling of the lips. As the strongest cause of CG is currently unknown, this unique case brings awareness to the infectious causes leading to CG. It calls for a greater need to investigate the reason behind certain pathogens specifically targeting the lips without causing any other systemic effects.

## Introduction

Cheilitis granulomatosa (CG) is also called Miescher cheilitis, a rare idiopathic inflammatory disorder characterized by persistent and progressive swelling of one or both lips [1,2]. Melkersson-Rosenthal syndrome (MRS) is a systemic granulomatous disease presenting most often with the triad of oral-facial edema, facial palsy, and fissures within the tongue [3]. CG is found to be the most common monosymptomatic form of MRS. If a patient only presents with lip swelling, it could indicate MRS even if they may not initially present with the other two triad symptoms, as the triad is not always present in the diagnosis of MRS [3]. CG is also under the umbrella term of orofacial granulomatosis, designated by Wiesenfeld et al. in 1985, for noninfectious conditions presenting with noncaseating granulomas in the face, oral cavity, and lips [4].

It can appear in any age group but is most prominent in the second or third decade of life. It is an extremely rare condition with limited cases currently reported in the literature. It is estimated that CG occurs in only 0.08% of the population [5]. The gender preference of CG has yet to be determined. Miest et al. and Al-Hamad et al. report no gender predilection; however, Mignogna et al. have reported a higher prevalence in females versus Lazzerini, who reported a higher prevalence in males [6-9]. CG most commonly affects the upper lip over the lower lip but can also affect the face, oral mucosa, pharynx, larynx, and gums. Symptoms can include a burning sensation, erythema, fissures, erosions, edema, and scaling [5,10,11].

Pathogenically, it is characterized by the presence of perilymphatic noncaseating granulomas, which include intralymphatic histiocytosis and lymphatic dilution [12]. Immunohistochemical studies have found a cluster of differentiation (CD) 68+ macrophages within the granulomas and CD163+ macrophages within the superficial lamina propria, interstitial areas, and perivascular lymphatics, indicating a type 1 helper (Th1) cell immune-mediated response [12].

Many cases of CG remain idiopathic with no identifiable causes. However, several potential causes have been proposed, including immunological factors, genetics, allergic reactions, and infectious agents [5]. Diagnosis is established based on the patient’s history, clinical presentation, and histopathological findings.

This paper presents the case of a 46-year-old male patient who presented with a chief complaint of upper and lower lip swelling and was found to be diagnosed with CG due to pathogenic causes of *Saccharomyces cerevisiae* and *Borrelia burgdorferi*.

## Case presentation

**Figure 1:**
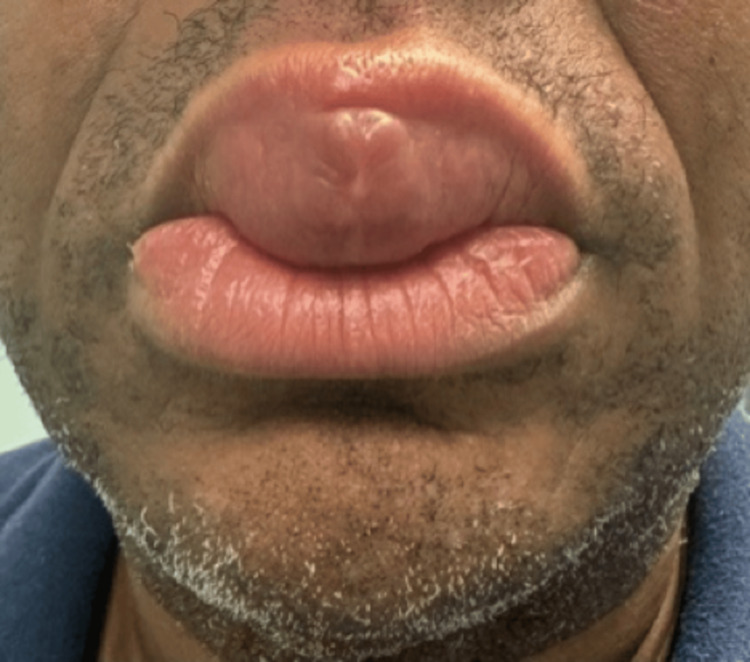
Frontal view of the patient’s lip swelling

**Figure 2:**
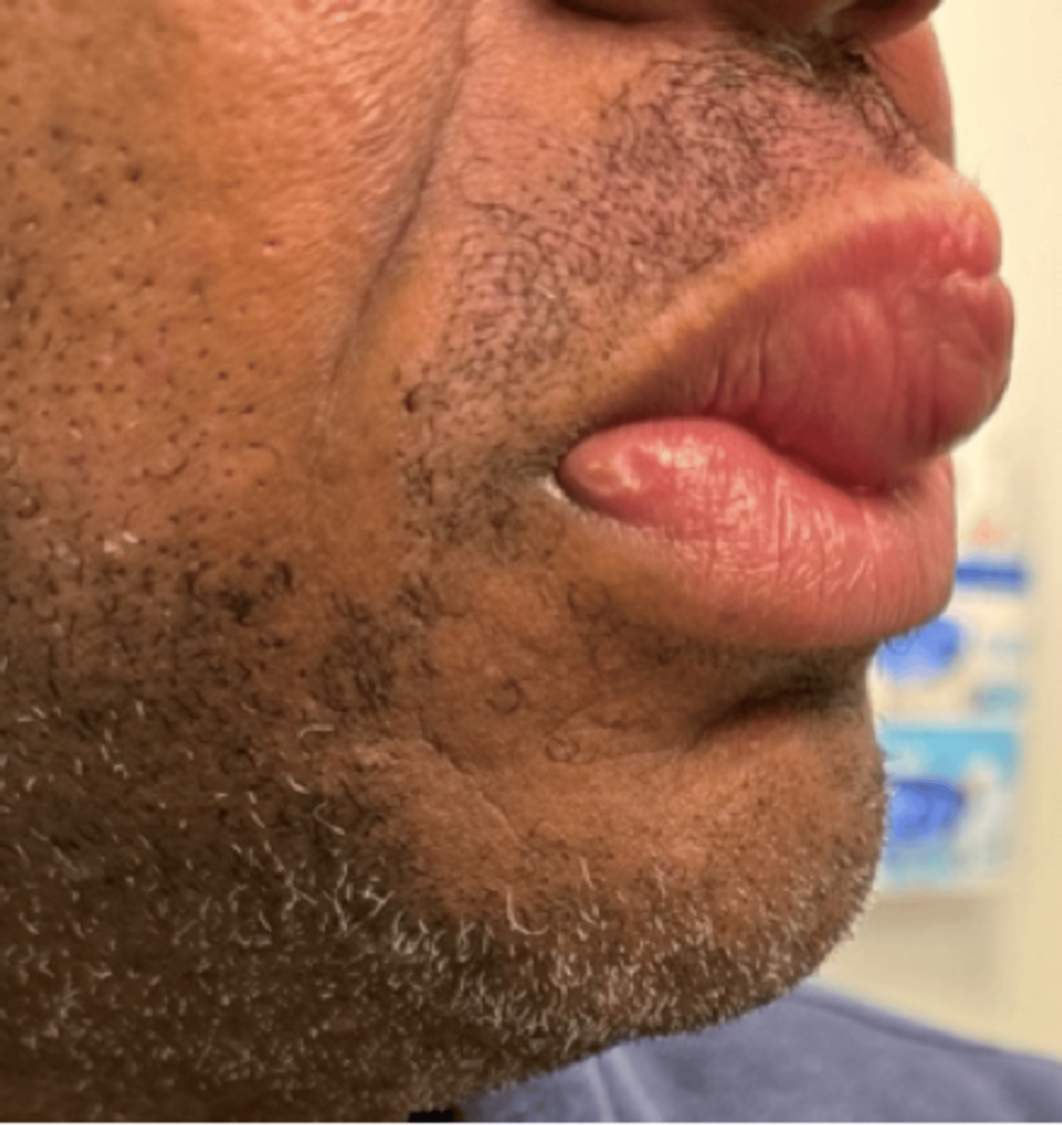
Side view of the patient’s lip swelling.

The patient is a 46-year-old male patient presenting to a rheumatology outpatient clinic with a chief complaint of progressive lip swelling since 2019 and left knee pain (Figures 1, 2). The patient was seen previously by an ear-nose-throat (ENT) specialist who performed a biopsy. The biopsy indicated edematous skeletal muscle, fibrovascular tissue with focal areas of chronic inflammation and noncaseating granulomas with multinucleated histiocytes. The biopsy was negative for acid-fast bacilli and fungal organisms. At the time, the patient was prescribed oral prednisone for the lip lesion, which reduced the swelling, but after discontinuing the steroids, the swelling returned, motivating the patient to seek further care. The patient has visited numerous other physicians, and no diagnosis was able to be made for his condition until this visit.

The patient has a medical history remarkable for hypertension and sleep apnea, with a family history of premature coronary artery disease in his father and previous breast cancer in his mother. In the patient’s review of systems, he denies chills, fatigue, fever, malaise, night sweats, weight gain, weight loss, nasal or ear drainage, hearing loss, sinus pressure, sore throat, tinnitus, vertigo, eye pain, eye discharge, vision loss, vision changes, cough, dyspnea, known tuberculosis exposure, chest pain, edema, palpitations, abdominal pain, change in bowel movements, nausea, vomiting, dysuria, hematuria, polyuria, incontinence, polydipsia, polyphagia, dizziness, weakness, headache, numbness, syncope, seizures, tremors, anxiety, depression, insomnia, brittle hair or nails, hives, purities, rash, skin lesions, back pain, joint pain, joint swelling, easy bruising or bleeding, and lymphadenopathy. The patient denied any previous tick bites or a history of Raynaud’s, ankylosis spondylitis, inflammatory bowel disease, psoriasis, enthesitis, dactylitis, uveitis, photosensitivity, and serositis. He denied any smoking and alcohol use. His medications include metoprolol 25 mg daily, losartan 100 mg daily, spironolactone 25 mg daily, and nifedipine 90 mg daily. The patient indicated he has no known allergies, with a recent allergy panel being completed on the patient. Vital signs in the initial appointment include a blood pressure of 124/86, temperature of 36.6 °C (97.8 F), heart rate of 74 beats per minute, 18 breaths per minute and 92% oxygen saturation. The physical exam findings revealed no abnormalities except for a posterior cyst and swelling in the left knee, as well as mild pain with motion. The patient was referred for a knee X-ray, an ultrasound to assess deep vein thrombosis (DVT), a chest computed tomography (CT) scan, and laboratory testing.

On the follow-up visit, imaging findings and laboratory results (Tables 1-3) were analyzed and discussed with the patient. Chest CT indicated no adenopathy, left knee X-ray revealed degenerative joint disease, and ultrasound was negative for DVT. Notably, in the laboratory results, the patient had negative results for rheumatological antibodies. Most significantly, the patient tested positive for Saccharomyces IgG and IgM, as well as four bands of Lyme disease, as reported by a previous provider. However, on repeat analysis, only the 58 kDa (IgG) band remained positive.

**Table 1:** Laboratory results for antibodies. IgA, immunoglobin A; IgG, immunoglobin G; IgM, immunoglobin M; DNA, deoxyribonucleic acid

Labs	Findings	Normal range
Antinuclear antibody	Negative	Titer <1:40
B2 glycoprotein (IgA, IgG, IgM)	<2.0 U/mL	<20 U/mL
Cardiolipin (IgA, IgG, IgM)	<2.0 U/mL	IgM < 12.5 U/mL; IgG <15 U/mL
Centromere B antibody	Negative	<0.1 Antibody Index
Anti-chromatin antibody	Negative	0-20 Units
Double-stranded DNA antibody	Negative	<10 IU/mL
Jo-1 antibody	Negative	0 Units
Rheumatoid factor (RF)	7 U/mL	<20 U/mL
Ribonucleoprotein antibody	Negative	<1 Unit
Scleroderma antibody-70	Negative	<1 Unit
Sjorgen’s antibody	Negative	<1 Unit/mL
Anti-Smith antibody	Negative	<7 Unit/mL
Antineutrophil cytoplasmic antibody	Negative	Titer <1:20
Myeloperoxidase antibody	<0.1 Antibody Index	0-0.9 Antibody Index
Proteinase-3 antibody	<0.1 Units	<0.4 Units
Angiotensin-1-converting enzyme	30 mcg/L	<40 mcg/L
C-reactive protein	5.0 mg/L	<3 mg/L
C1 esterase inhibitor	30 mg/dL	15-30 mg/dL
Histone antibody	<1.0 Units	<1 Units
Human Leukocyte Antigen B27	Negative	Negative
Lyme antibody 18KD (IgG) Band	Non-reactive	Non-reactive
Lyme antibody 23KD (IgG) Band	Non-reactive	Non-reactive
Lyme antibody 23KD (IgM) Band	Non-reactive	Non-reactive
Lyme antibody 28KD (IgG) Band	Non-reactive	Non-reactive
Lyme antibody 39KD (IgG) Band	Non-reactive	Non-reactive
Lyme antibody 39KD (IgM) Band	Non-reactive	Non-reactive
Lyme antibody 41KD (IgG) Band	Non-reactive	Non-reactive
Lyme antibody 41KD (IgM) Band	Non-reactive	Non-reactive
Lyme antibody 45KD (IgG) Band	Non-reactive	Non-reactive
Lyme antibody 58KD (IgG) Band	Reactive	Non-reactive
Lyme antibody 66KD (IgG) Band	Non-reactive	Non-reactive
Lyme antibody 93KD (IgG) Band	Non-reactive	Non-reactive
Lyme disease AB (IgG)	Negative	Negative
Lyme disease AB (IgM)	Negative	Negative
Parvovirus B19 antibody	1.5 Antibody Index	<0.9 Antibody Index
Saccharomyces cerevisiae IgG *antibody*	36.2	<20 Units
Saccharomyces cerevisiae IgA *antibody*	36.3	<20 Units
Hepatitis B core antigen	Non-reactive	Non-reactive
Hepatitis B surface antigen	Non-reactive	Non-reactive
Hepatitis C antibody	Non-reactive	Non-reactive

**Table 2:** Laboratory results for complete blood count.

Labs	Findings	Normal range
Red blood cell	4.47 million µL	Males: 4.7-6.1 million µL
White blood cell	8.2 × 10^3 µL	4.8-10.8 × 10^3 µL
Platelet	280 × 10^3 µL	130-400 × 10^3 µL
Neutrophils	58.9%	41%-77%
Mean platelet volume	10 fL	7.5-11.5 fL
Mean corpuscular hemoglobin	29.8 pg/cell	27-31 pg/cell
Mean corpuscular volume	86.8 fL	80-100 fL
Basophils	0.9%	0-1%
Eosinophils	2.9%	0-3%
Lymphocytes	29.7%	24%-44%
Hematocrit	38.8%	42%-52%
Hemoglobin	13.3 g/dL	14-18 g/dL

**Table 3:** Laboratory results for complete metabolic panel.

Labs	Findings	Normal range
Albumin	3.9 g/dL	3.4-5.4 g/dL
Alanine aminotransferase	18 U/L	4-36 U/L
Aspartate aminotransferase	15 U/L	8-33 U/L
Bilirubin	0.9 mg/dL	0.1-1.2 mg/dL
Calcium	9.3 mg/dL	8.5-10.2 mg/dL
Carbon dioxide	28 mEq/L	23-29 mEq/L
Chloride	105 mEq/L	96-106 mEq/L
Creatinine	1.38 mg/dL	0.6-1.3 mg/dL
Estimated glomerular filtration rate	64 mL/minute/1.73 m^2	60-89 mL/minute/1.73 m^2
Globulin	4.0	2.3-3.4 g/dL
Glucose	85 mg/dL	70-100 mg/dL
Potassium	3.8 mEq/L	3.7-5.2 mEq/L
Sodium	139 mEq/L	135-145 mEq/L
Protein	7.9 g/dL	6-8.3 g/dL
Blood urea nitrogen (BUN)	16 mg/dL	6-20 mg/dL
BUN/creatinine ratio	12:1	10-20:1

Following the positive results for* Saccharomyces cerevisiae* and Lyme disease caused by *Borrelia burgdorferi* in addition to histopathology indicating noncaseating granulomas of the lips, a diagnosis of CG was made. The patient was started on immunosuppressive therapy with mycophenolate. Per the patient’s request for surgical intervention to remove the lip lesion, he was referred to an ENT specialist. He was also referred to an orthopedic specialist for knee pain.

**Figure 3:**
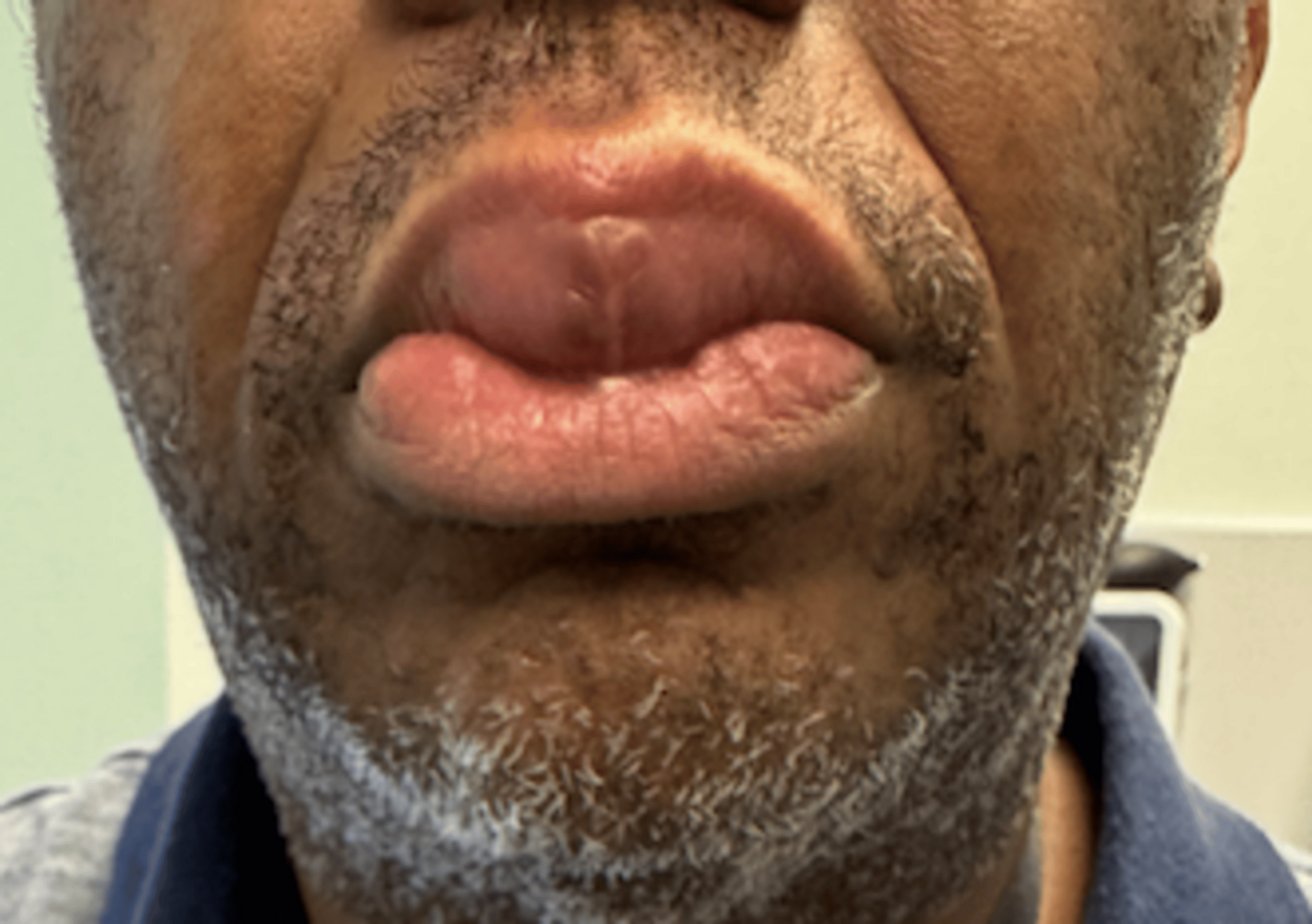
Frontal view of the patient’s lip following treatment with mycophenolate.

**Figure 4:**
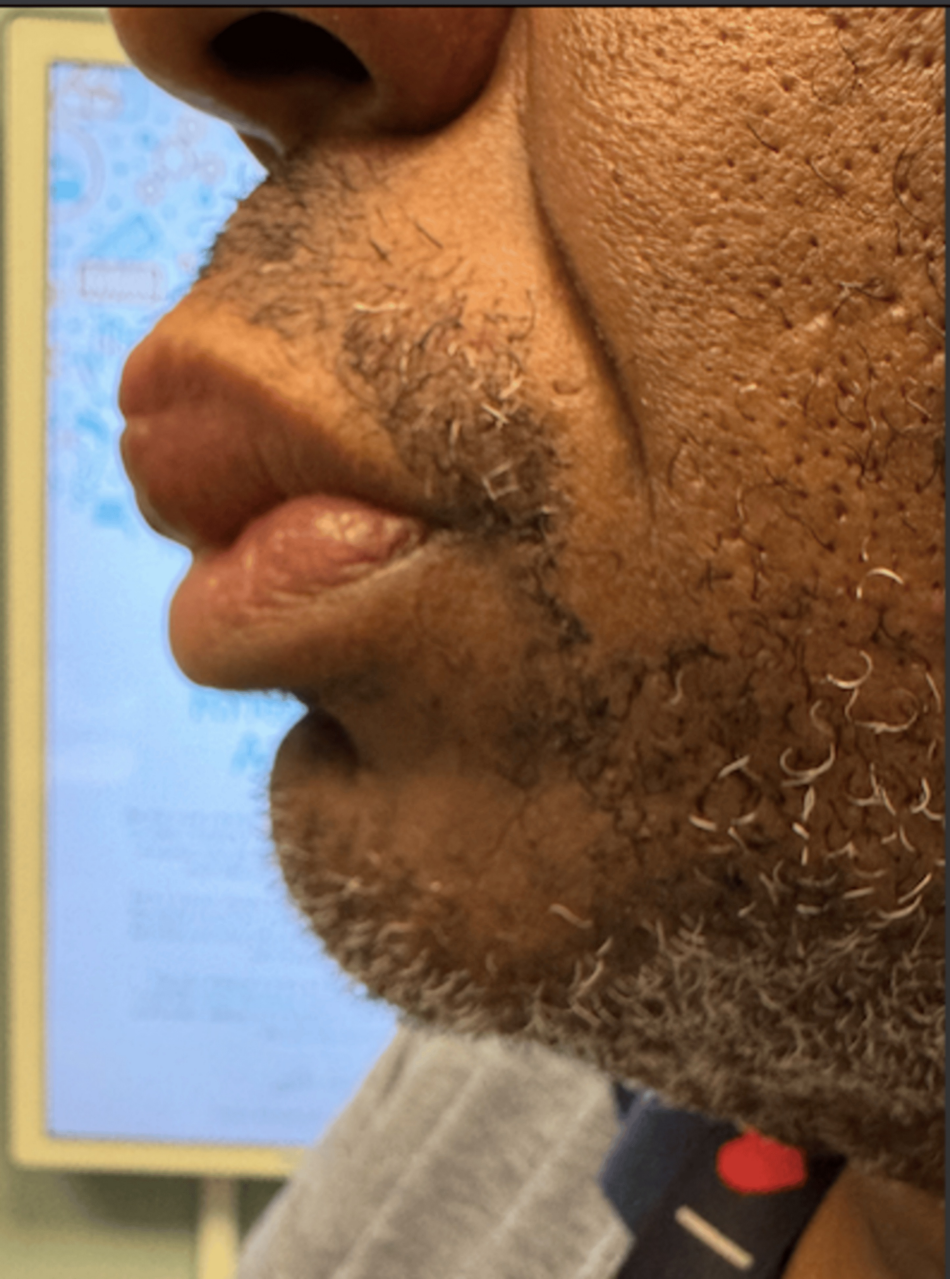
Side view of the patient’s lip following treatment with mycophenolate.

The patient returned for follow-up to the clinic after initiation of mycophenolate and had a noticeable reduction of lip swelling (Figures 3, 4). The patient’s approval and consent for the publication of this case report was received.

## Discussion

CG is a rare and poorly understood condition, estimated to affect only 0.08% (8 out of 10,000) of the general population, primarily between the ages of 20 and 40 [1-3]. The etiology of CG is currently unknown, with hypotheses linking it to various factors, including genetics, allergies, immunological processes, and infections. Genetic origin has been considered if numerous family members present with CG; however, no concrete genetic link has been found. This patient had no family history of a similar event. Allergic responses have also been considered; however, this relationship has not been well established in the literature. This patient recently underwent a previous allergy panel to screen for any allergies, but all findings were negative. Immunologically, CG is a Th1-mediated response, and it is thought that an alteration within the innate immunity of the lip mucosa to specific antigens can trigger a response leading to non-caseating granulomas. Finally, microbial factors, including *Mycobacterium tuberculosis*, *Mycobacterium avium*, *Borrelia burgdorferi,* *Saccharomyces cerevisiae*, and *Candida albicans*, have been considered as culprit agents. However, these associations have yet to be proven in the literature until this patient case [1]. Our patient tested positive for Saccharomyces cerevisiae and four bands of Lyme disease. We hypothesize that an infectious process is the primary culprit in this patient’s CG development. It is also important to note the connection between CG and Chron’s disease, with CG being found in almost 1% of these patients, it is thought to be an extra-intestinal presentation of the disease [1]. However, this patient had no evidence of Chron’s disease, which was ruled out through analysis of past medical history and symptomology. 

Two histopathologies have been described for CG. Biopsy of patients with CG can present either type or a combination of both. The first, typically the earlier presentation of CG, is the lymphedematous type, characterized by lymphatic dilation, lymphedema, and plasma cell infiltration. The later presentation is the sarcoid type, marked by non-caseating granulomas composed of epithelioid histiocytes, lymphocytes, plasma cells, macrophages, and edema within the interstitial connective tissue [13,14]. CD68+ macrophages are the most prominent within CG, indicating Th1 immune response, which entails increased levels of interferon-gamma, interleukin (IL)-12, C-C chemokine receptor 5, and C-X-C chemokine receptor 3 that promote macrophage polarization to induce a Th1 response against pathogens, which induces epithelioid granulomas [15,16]. The elevation of CD68+ macrophages being identified more commonly lends the diagnosis of CG to lean more toward a microbial etiology. It is hypothesized that the macrophages can spread through the lymphatic channels, leading to lymphatic and tissue edema, which results in visible lip swelling. As well, macrophages in the form of histocytes can spread to the surrounding area to form extravascular granulomas [13-15]. Further research is required to understand better the Th1 response of CG, and potential targets within this pathway that can be used for treatment.

To our knowledge, there has not been a case reported where the infectious cause leading to CG was *Saccharomyces cerevisiae* and/or *Borrelia burgdorferi*. While both agents have been recognized in the literature as potential causative factors, no case studies have yet established a direct connection [12].

*Saccharomyces cerevisiae* is a unicellular fungus known as baker’s yeast or brewer’s yeast, commonly used in foods, wines, and beers. Digestive colonization often occurs following ingestion. *Saccharomyces cerevisiae* is emerging as an opportunistic agent in severely immunocompromised patients and has been found to lead to pneumonia, endocarditis, liver abscess, fungemia, and sepsis. *Saccharomyces cerevisiae* antibodies have been found in the serum of patients with chronic granulomatous conditions such as tuberculosis, Chron’s disease, and sarcoidosis. Patients with orofacial granulomatosis with elevated *Saccharomyces cerevisiae* IgA levels have been found to have a higher chance of being associated with gastrointestinal issues [17]. There is a potential connection between *Saccharomyces cerevisiae* as the causative agent of this patient’s CG. However, its presence can also indicate a disruption of the gut barrier associated with an inflammatory condition, though this patient exhibited no symptoms to support that explanation.

*Borrelia burgdorferi *is the pathogenic spirochete responsible for Lyme disease via a tick bite. Symptoms of Lyme disease include an annular rash, arthritis, carditis, and encephalopathy [18]. This patient declines any travel outside of the state of Florida and declines any trips to a wooded area or a previous tick bite. It is an interesting finding that the patient had four bands positive for Lyme disease without the traditional risk factors. Previous studies have been unable to find a connection between CG and *Borrelia burgdorferi* until potentially, this case study [12].

This raises questions on whether both *Saccharomyces cerevisiae* and *Borrelia burgdorferi *led to this patient’s CG or if one has a greater influence leading to its development; however, we are unaware of the timing between the patient's infection with these agents and the initiation of the lip swelling.

Differential diagnoses to be considered when evaluating a patient with similar lip swelling commonly is angioedema with other options including contact dermatitis, granular cheilitis, oral abscess, sarcoidosis, tuberculosis, and adverse reaction to medications such as angiotensin-converting enzymes inhibitors and nonsteroidal anti-inflammatory medications.

Diagnosis of CG is largely based on clinical findings, and once a patient presents with markedly swollen lips, a good history is required to be taken, complete labs, and a biopsy [1,3]. Once a diagnosis is established based on histopathology, it is crucial to rule out autoimmune causes by conducting comprehensive autoimmune blood panels. Infectious causes should also be investigated through laboratory testing, chest radiography, and a Mantoux test to exclude other granulomatous diseases such as tuberculosis and sarcoidosis. Additionally, an ultrasound of the abdomen and pelvis should be performed to rule out Crohn’s disease, as a link between CG and Crohn’s has been suggested.

The current treatment for CG is challenging, and it requires a combination of treatments. The classic treatment that has been tested is corticosteroid therapy to reduce inflammation [19,20]. This was previously trailed in our patient; however, it did not maintain a reduction in swelling. Nonsteroidal systemic agents have been used including clofazimine, hydroxychloroquine, and sulfasalazine to avoid long-term corticosteroid-negative effects [21]. Other treatments that have been used in cases include anti-inflammatory or immunosuppressive medications, including methotrexate, fumaric acid esters, infliximab, and thalidomide; however, none have shown consistent findings [22]. Immunotherapy has recently been tested within this population. Treatment of CG with ustekinumab, targeting IL-23, was deemed to be effective following four months of treatment [23]. Furthermore, antibiotics, including minocycline, roxithromycin, and metronidazole, have also been tested, but due to no infectious agent being officially linked to CG, the benefit of these medications is more geared towards their anti-inflammatory and immune modulation [20]. Surgery is reserved for cases that cause significant disfiguration. Surgically, cheiloplasty reduction can be considered when the lesion is fixed and stable to improve function and cosmetic appearance, often resulting in complete remission [24]. Helium-neon laser radiation has also been tested in some patients, showing benefits when the disease duration is less than four years [1,21,25].

CG is a long-term benign condition that varies between flare-ups and moments of improvement. The more progressive and severe flare-ups the patient experiences, the more likely they are to have irreversible damage to their lips. It is important to note that as it is a benign condition, no direct impacts on life span have been found. However, patients should be cautioned that CG may be a part of MRS to watch for signs of facial paralysis in the future, as well as any changes in bowel function, as it may be linked to Chron’s disease [1].

This case exemplifies a strong infectious cause behind the development of CG. Further large studies should be conducted to analyze pathogenic findings within patients to further our understanding of which microbes are more or less likely to lead to CG.

## Conclusions

CG is a rare and often challenging condition to diagnose and manage, given its variable clinical presentation and potential overlap with other systemic diseases. This case highlights the importance of a thorough clinical evaluation, including a detailed history, comprehensive physical examination, and appropriate investigations such as histopathology and imaging, to establish an accurate diagnosis. This patient case provides stronger evidence for the infectious cause hypothesis as the leading trigger of CG. Further research is needed to elucidate the pathogenesis of CG and explore more effective therapeutic options for long-term disease control.
